# Milling Parameter Optimization of Continuous-Glass-Fiber-Reinforced-Polypropylene Laminate

**DOI:** 10.3390/ma15072703

**Published:** 2022-04-06

**Authors:** Hanjie Hu, Bing Du, Conggang Ning, Xiaodong Zhang, Zhuo Wang, Yangyang Xiong, Xianjun Zeng, Liming Chen

**Affiliations:** 1 School of Aeronautics, Chongqing Jiaotong University, Chongqing 400074, China; huhj@gatri.cn; 2 The Green Aerotechnics Research Institute of CQJTU, Chongqing 401120, China; zengxj@gatri.cn; 3 Chongqing Key Laboratory of Nano–Micro Composite Materials and Devices, School of Metallurgy and Materials Engineering, Chongqing University of Science and Technology, Chongqing 401331, China; 2018441648@cqust.edu.cn (C.N.); 2018441447@cqust.edu.cn (X.Z.); 2018444415@cqust.edu.cn (Z.W.); 2019442412@cqust.edu.cn (Y.X.); 4 College of Aerospace Engineering, Chongqing University, Chongqing 400030, China

**Keywords:** continuous glass fiber, thermoplastic composite, milling, process parameter

## Abstract

The composite-material laminate structure will inevitably encounter connection problems in use. Among them, mechanical connections are widely used in aerospace, automotive and other fields because of their high connection efficiency and reliable connection performance. Milling parameters are important for the opening quality. In this paper, continuous-glass-fiber-reinforced-polypropylene (GFRPP) laminates were chosen to investigate the effects of different cutters and process parameters on the hole quality. The delamination factor and burr area were taken as the index to characterize the opening quality. After determining the optimal milling tool, the process window was obtained according to the appearance of the milling hole. In the selected process parameter, the maximum temperature did not reach the PP melting temperature. The best hole quality was achieved when the spindle speed was 18,000 r/min and the feed speed was 1500 mm/min with the corn milling cutter.

## 1. Introduction

The composite-material laminate structure will inevitably encounter connection problems in use. Among them, mechanical connections are widely used in aerospace, automotive and other fields because of their high connection efficiency and reliable connection performance [1,2,3]. The drilling process is an indispensable process. During the processing, there are defects such as fiber pullout, tearing, peeling, delamination and burrs, which seriously affect the mechanical properties of the opening holes, further affecting the performance of composite structures. Therefore, it is of great research value to find a method that can reduce the occurrence of defects and improve the quality of holes [4].

Prasad and Chaitanya [5] used the Taguchi analysis method to study the delamination factors of laminates and optimized the processing parameters. The experimental results show that the peeling delamination is most affected by the thickness of the material, followed by the feed speed and fiber orientation, and the delamination rate is most affected by feed speed, followed by thickness, fiber orientation and spindle speed. Based on the numerical analysis of drilling images, Hrechuk et al. [6] carried out the non-destructive quantification of visible defects and proposed an overall parameter Q. The results of drilling experiments show that the drilling quality Q, the number of drillings, and the tool wear are linearly related. Liu et al. [7]. proposed a new delamination scheme in which delamination caused by thrust exerted by the chisel and cutting edges is considered. Amaro et al. [8] found that the presence of holes increased the energy absorbed by the damaged and delaminated regions. The delamination distribution is also affected due to the change in the interlayer shear-stress distribution, which is the main reason for the delamination between layers with different orientations. Tsao et al. [9] proposed a new method for the equivalent delamination factor and compared it with the adjusted delamination factor and the conventional delamination factor, which is suitable for characterizing delamination at the hole exit after drilling composites. Sugita et al. [10] proposed the design of a drilling tool that is shaped to suppress burrs and delamination during composite drilling. Loja et al. [11] used four different types of tools to conduct drilling experiments on glass-fiber laminates to analyze and evaluate the delamination effect and its maximum temperature. The experiments showed that the higher the drilling speed, the higher the temperature generated by friction, and the greater the damage to the aperture diameter. Velaga et al. [12] used a double-flute twist drill with a diameter of 10 mm to conduct drilling experiments on epoxy-resin/glass-fiber composites. The experimental results found that the rotation speed of 700 rpm and the feed rate of 0.4 mm/rev were used for the optimum drilling parameters of the selected tool. Guo [13] conducted cutting experiments on continuous-glass-fiber-reinforced-polypropylene composites at different cutting angles to analyze the characteristics of right-angle cutting and the causes of damage. The experiment found that the fiber cutting angle has a significant impact on the cutting process: at 0° there is groove damage; at 45° the cutting surface is the best; at 90° there is a slight burr; at 135° the resin interface cracks and many burrs are generated. Mustari et al. [14]. investigated the effect of glass-fiber content in glass-fiber composites on the drilling process. The results show that the drilling surface quality is closely related to the percentage of fiber content in the sample. In terms of roundness deviation, delamination, fiber pull-out and taper angle, the composite samples performed better when the glass-fiber content was lower. Patel et al. [15] aimed to examine the effect of tool geometry, spindle speed and feed on thrust and delamination in hybrid hemp-glass composites. Palanikumar et al. [16] studied the parameters affecting the thrust of glass-fiber-reinforced polypropylene (GFRPP) in boreholes and established an empirical relationship for determining the thrust of GFRTP drilling. Srinivasan et al. [17] used the “Brad and Spur” drill bit to drill the GFRPP matrix composites with the aim of testing the effect of drilling parameters on the roundness error, using the Box-Behnken design (BBD) technique to determine the cutting parameters that were evaluated and optimized. The results show that the model can be effectively used to predict the response variable and thus control the roundness error. Palanikumar et al. [18] studied the effect of various feed rates on thrust, spindle speed, and bit geometry using Brad and straight plunger bits, and the experimental results showed that delamination is entirely dependent on the feed rate.

In this paper, GFRPP laminates are taken as the research object to investigate the effects of different cutters and process parameters on the hole quality. The delamination factor and burr area were taken as the index to characterize the opening quality. After determining the optimal milling tool, the process window was obtained according to the appearance of the milling hole. Afterwards, the response surface method (RSM) was utilized to analyze the influence of the process parameters on the opening quality.

## 2. Materials and Methods

**Figure 1:**
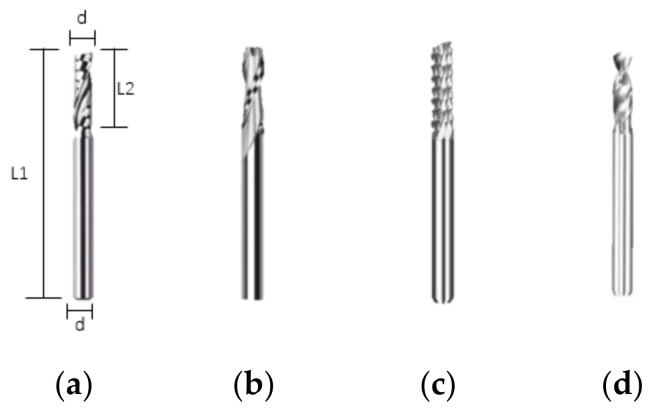
Schematic of cutting tools: (**a**) Single-edge milling cutter; (**b**) Double-edge milling cutter; (**c**) Corn milling cutter; (**d**) PCB alloy drill.

This experimental sample was made of GFRPP prepreg in order to prepare composite material laminate, and glass-fiber-reinforced-polypropylene prepreg with a thickness of 0.3 mm was fiber reinforced using [0/45/-45/90]s. Composite laminate, milled to 2.4 mm through holes using the hot press (Qingdao Huabo Machinery Technology Co., Ltd., Qingdao, China), according to the process parameters in [19]. Milling experiments were conducted by a CNC (Computer Numerical Control) engraving machine (JingYan Instruments & Technology CO., Ltd., Dongguan, China). As shown in Figure 1, milling tools included a single-edge spiral milling cutter, double-edged spiral milling cutter, corn milling cutter and PCB (Printed Circuit Board) alloy drill with the same size where *L*_1_ = 38 mm, *L*_2_ = 12 mm, *d* = 3.175 mm. First, the comparison among milling tools was made under the same spindle speed and feed speed of 6000 r/min and 800 mm/min, respectively. Five holes with a diameter of 8 mm were milled by each tool. The visual-inspection method was used to choose the optimal mill tool based on the macroscopic appearance near the hole.

In the milling process of traditional composite laminates, the local thermal shock [20] caused by the presence of abrasives and fibers and the low thermal conductivity of composite materials limit the temperature dissipation during hole opening, resulting in heat accumulation at the hole exit and excessively high temperature, and even in the case of improper heat dissipation treatment, the temperature will often be higher than the glass transition temperature, resulting in a decrease in the performance of the resin matrix and a significant reduction in the quality of openings [21]. In the milling experiment, the temperature change is often mainly affected by the spindle speed and feed rate [22]. Due to the wide selection of parameters in this experiment, the effect of temperature on the quality of the opening cannot be excluded from the experimental results.

**Figure 2:**
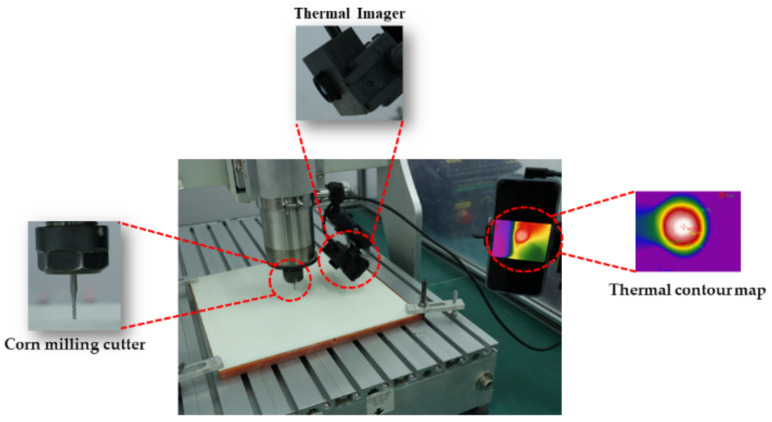
Thermal-imaging setup.

**Figure 3:**
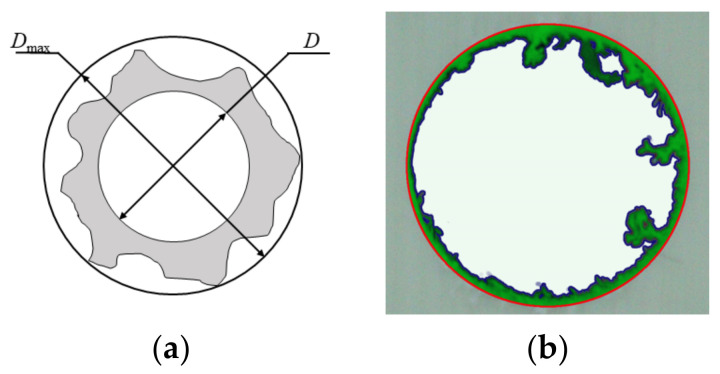
Schematic diagram of evaluation parameters: (**a**) Delamination factor; (**b**) Burr area.

Therefore, a real-time temperature-observation platform was built, and special parameter-processing points were selected to study the effect of temperature changes on the quality of the opening during milling, as depicted in Figure 2. The infrared thermal imager was supported and fixed to the main shaft of the cutting machine by a movable bracket and was able to move on the same trajectory with the main shaft of the cutting machine. The thermal-imaging camera directly captures the milling hole, and is connected to an external receiving software device, which can display the milling temperature change on the screen in real time. A Canon digital camera (EOS 200DⅡ, Canon (CHINA) CO., Ltd., Beijing, China) was used to take pictures of the openings, and ImageJ and Photoshop software were used to process the pictures to calculate the delamination factor and burr area during milling, as shown in Figure 3. The delamination factor $F_{d}$ was defined as the actual diameter divided by the designed diameter, referring to Equation (1). The delamination factor and burr area were taken as the index to characterize the opening quality. After determining the optimal milling tool, the process window was obtained according to the appearance of the milling hole. Afterwards, the response surface method (RSM) was utilized to analyze the influence of the process parameters on the opening quality.

(1) $$F_{d}=\frac{D_{max}}{D}$$

where *D* is the designed diameter of the hole and *D_max_* is the actual diameter.

## 3. Results

### 3.1. Determining the Process Window

**Figure 4:**
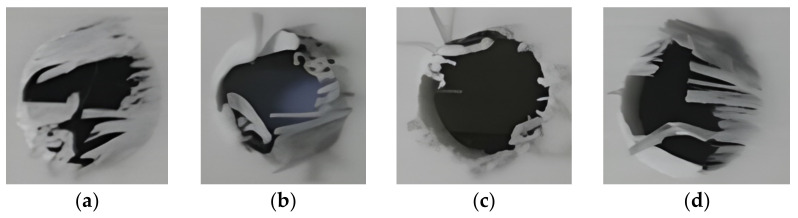
Morphology of milling holes: (**a**) Single-edge milling cutter; (**b**) Double-edge milling cutter; (**c**) Corn milling cutter; (**d**) PCB alloy drill.

As seen in Figure 4, under the spindle speed of 6000 r/min and feed speed 800 mm/min, the four milling tools had different defects. The single-edged milling cutters, double-edged milling cutters, and PCB alloy drill had many burrs and serious fiber delamination. For the corn milling cutter, no visual severe delamination was found and relatively less burrs appeared compared with the other three tools. Loja et al. [11] used four drilling tools to evaluate the delamination and maximum temperature caused by drilling in fiberglass laminates. For milling cutters and twist drills, the higher the drilling speed, the higher the temperature generated by friction, and the greater the damage to the aperture. The experimental results and literature data have certain commonality. The quality of the milling effect of the corn milling cutter was better than that of the spiral milling cutter and twist drill. The subsequent experiment was conducted by the corn milling cutter.

**Figure 5:**
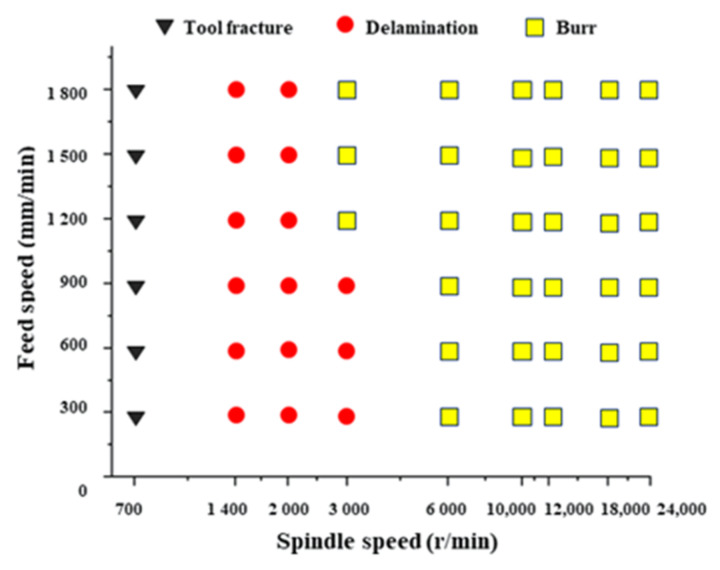
Influence of process parameters on milling property.

**Figure 6:**
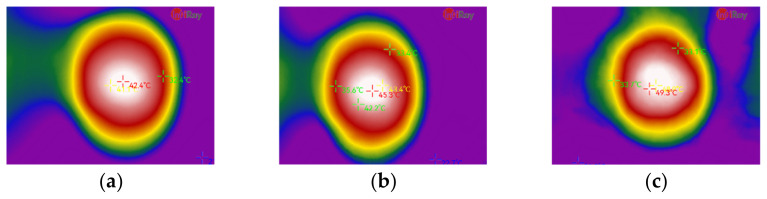
Thermal contour map of milling hole: (**a**) F1-S2; (**b**) F3-S5; (**c**) F6-S9.

In this step, spindle speed and feed speed were varied from 700 r/min to 24,000 r/min and 300 mm/min to 1500 mm/min, respectively, as listed in Table 1. A total of 27 groups of experiments were made and two holes were milled under each group of parameters to ensure the results. The influence of the process parameters on milling property is shown in Figure 5, where three typical situations known as tool fracture, delamination and burr were found. When the spindle speed was too low, 700 r/min, tool fracture appeared. When the spindle speed was lower than 3000 r/min, the delamination of fibers near the hole was found. On the contrary, the burr became dominant when the spindle speed was higher than 6000 r/min. Between the range of 3000 r/min to 6000 r/min was the transition zone where delamination and burr coexisted. From Figure 6, three combinations of processing parameters named F1–S2, F3–S5, and F6–S9 were selected as the thermal-imaging objects. It was shown in Figure 6 that with the increase in the spindle speed and feed speed, the maximum temperature increases, but the maximum temperature was 49.3 °C below the melting temperature of PP.

### 3.2. Optimizing the Process Parameters

Based on the above process window, the process parameters were refined. Feed speed was chosen as 1200 mm/min, 1500 mm/min, and 1800 mm/min, and the spindle speed was chosen as 12,000 r/min, 18,000 r/min, and 24,000 r/min. The response-surface-method analysis was carried out to determine the optimal process parameters. The factor-level design table is shown in Table 2 with two factors and three levels. The experimental trials were design as in Table 3 with the corresponding characterization variables named delamination factor and burr area. It can be seen that with the change in the processing parameters, the delamination factor is the lowest at 1.0046 and the highest at 1.0374. Taking the delamination factor as the dependent variable and the specific level of each factor as the independent variable, the quadratic regression equation was established to obtain the following results as Equation (2):

(2) $$F_{d}=0.003887A^{2}+0.002667B^{2}-0.005302AB-0.009877A-0.005397B+1.02$$

The significance analysis of each item in the quadratic regression equation is shown in Table 4. It shows that under the selected experimental interval, the *F* value of this model is 15.21628 and the probability of data error is 1.04%. The analysis of variance for the model is shown in Table 5, showing that 95% of the experimental data can be explained by this model. Adj *R*^2^ and Pred *R*^2^ had higher values and the difference was within 0.2, indicating that the regression model can fully fit the test results. However, Table 6 shows that the relationship between burr area and the model was not significant, although the burr area decreased with the increase in the processing parameters.

From the above, it can be concluded that when the machining parameters were low, the hole quality was not ideal. With the increase in the spindle speed and feed speed, the hole quality was gradually improved. When the machining parameters were increased to a certain range, the effect of improving the quality of the opening became weaker. The results of the response surface method show that when the spindle speed was 18,000 r/min and the feed speed was 1500 mm/min, the hole quality was the best, and the delamination factor and the burr area were both the minimum.

## 4. Conclusions

In this paper, GFRPP laminates were taken as the research object to investigate the effects of different cutters and process parameters on the hole quality. The specific conclusions are as follows:

(1) Different tools have a great influence on the hole quality, and the corn milling cutter used for GFRPP laminates has the best opening quality.
(2) In the selected region, with the increase in spindle speed and feed speed, the change in temperature distribution in the entry surface near the hole is not significant, and the maximum temperature does not reach the resin-melting temperature.
(3) Using the delamination factor and burr area to represent the hole quality is feasible. The quadratic regression model is significant for delamination factor while insignificant for burr area. The reason is because that the calculation of the burr area is greatly affected by the experimental error.
(4) In the milling of presented GFRPP laminate, the best hole quality is achieved when the spindle speed is 18,000 r/min and the feed speed is 1500 mm/min with the corn milling cutter.

## Figures and Tables

**Table 1:** Number of process parameters.

Feeding Speed mm/min	No.	Spindle Speed r/min	No.
300	F1	700	S1
600	F2	1400	S2
900	F3	2000	S3
1200	F4	3000	S4
1500	F5	6000	S5
1800	F6	10,000	S6
		12,000	S7
		18,000	S8
		24,000	S9

**Table 2:** Factor-level design table of response surface method.

Level	*A*: Feeding Speed mm/min	*B*: Spindle Speed r/min
−1	2000	300
0	10,000	900
1	18,000	1500

**Table 3:** Design and test results of response surface method.

No.	*A*: Spindle Speed mm/min	*B*: Feeding Speed r/min	Delamination Factor	Burr Area mm^2^
1	2000	300	1.0352	15.17
2	18,000	300	1.0237	23.67
3	2000	1500	1.0374	25.07
4	18,000	1500	1.0046	8.94
5	2000	900	1.0322	30.94
6	18,000	900	1.0173	17.54
7	10,000	300	1.0313	20.67
8	10,000	1500	1.0158	12.99
9	10,000	900	1.0179	21.11
10	10,000	900	1.0179	24.49

**Table 4:** Significance analysis of delamination factor.

Source	Sum of Squares	*d*_f_	Mean Square	*F*-Value	*p*-Value Prob > *F*	
Model	0.000934	5	0.000187	15.21628	0.0104	significant
*A*–Spindle speed	0.000585	1	0.000585	47.66917	0.0023	
*B*–Feeding speed	0.000175	1	0.000175	14.23207	0.0196	
*AB*	0.000112	1	0.000112	9.159819	0.0389	
*A* ^2^	3.53 × 10^−5^	1	3.53 × 10^−5^	2.871886	0.1654	
*B* ^2^	1.66 × 10^−5^	1	1.66 × 10^−5^	1.352159	0.3095	
Residual	4.91 × 10^−5^	4	1.23 × 10^−5^			
Lack of Fit	4.91 × 10^−5^	3	1.64 × 10^−5^			

**Table 5:** ANOVA results of delamination factor.

*R* ^2^	Adj *R*^2^	Pred *R*^2^	Adeq Precisior
0.9501	0.8876	0.5152	11.254

**Table 6:** Significance analysis of burr area.

Source	Sum of Squares	*d*_f_	Mean Square	*F*-Value	*p*-Value Prob > *F*	
Model	335.7867	5	67.15734	5.901067	0.0551	not significant
*A*–Spindle speed	73.70933	1	73.70933	6.476785	0.0636	
*B*–Feeding speed	26.07555	1	26.07555	2.29124	0.2047	
*AB*	151.5951	1	151.5951	13.32055	0.0218	
*A* ^2^	4.637278	1	4.637278	0.407474	0.5580	
*B* ^2^	84.00382	1	84.00382	7.381355	0.0532	
Residual	45.52217	4	11.38054			
Lack of Fit	39.81506	3	13.27169	2.325465	0.4412	not significant

## Data Availability

The data presented in this study are available on request from the corresponding author.
